# Inverse Relationship between Serum Lipoxin A4 Level and the Risk of Metabolic Syndrome in a Middle-Aged Chinese Population

**DOI:** 10.1371/journal.pone.0142848

**Published:** 2015-11-13

**Authors:** Dan Yu, Zhiye Xu, Xueyao Yin, Fenping Zheng, Xihua Lin, Qianqian Pan, Hong Li

**Affiliations:** Department of Endocrinology, Sir Run Run Shaw Hospital Affiliated to School of Medicine, Zhejiang University, Hangzhou, Zhejiang, P. R. China; University of Maryland, UNITED STATES

## Abstract

Metabolic syndrome (MetS) has been identified to be associated with a state of chronic, low-grade inflammation in adipose tissue. Lipoxins are endogenously generated from arachidonic acid, and exhibit anti-inflammatory actions. Currently, there is no available cohort study identifying the association between serum lipoxins level and MetS. Here we investigate the relationship between serum lipoxin A4 (LXA4) level and the risk of incident MetS in a middle-aged Chinese population. A total 624 participants aged 40–65 years were enrolled at baseline, with 417 (including 333 MetS absence) of them were followed up at 2.5 years. Abdominal visceral fat area (VFA) and abdominal subcutaneous fat area (SFA) were determined using MRI. Serum lipoxin A4 levels were measured by ELISA. At baseline, serum LXA4 levels were significantly correlated with a cluster of traditional MetS risk factors related to obesity (P≤0.05). A higher incidence of new Mets was found in the participants of the lowest tertile of LXA4 levels as compared with that in participants of the highest tertile (P = 0.025). Low serum LXA4 levels [OR 2.607(1.151–5.909), P = 0.022] and high VFA [OR 2.571(1.176–5.620), P = 0.018] were associated with an increased incident Mets, respectively, which remained statistically significant after adjustment for age, gender, current smoking, and alcohol drinking status. Logistic regression analysis suggested a combination of low serum LXA4 levels and high WC/VFA might optimize the prediction of incident Mets in middle-aged Chinese population [OR 4.897/4.967, P = 0.009/0.003]. Decrease in serum LXA4 level and increase in VFA are independent predictors of incident Mets in a population-based cohort, and a combination of them enhances the prognostic value of incident Mets. Taken together, our data suggest that serum LXA4 levels might be useful for early detection and prevention of Mets.

## Introduction

Metabolic syndrome (MetS) has received increased attention in the past decade. Patients with MetS are at increased risk for developing type 2 diabetes mellitus (T2DM) and atherosclerotic cardiovascular disease. Decreased insulin sensitivity is the central feature of this syndrome. This syndrome has been noted to be associated with a state of chronic, low-grade inflammation, in which macrophages accumulate in adipose tissue and secrete inflammatory cytokines. Adipose inflammation is considered to be associated with insulin resistance[1–3]. The available evidence supports the concept that targeting inflammation improves insulin sensitivity and β-cell function; it also ameliorates glucose control in patients with MetS or T2DM[4, 5].

The resolution of inflammation was generally thought to be a passive process; however, it has recently been noted that an active process controlled by endogenous mediators with selective actions on inflammatory cells is also involved[6]. Pro-resolving lipid mediators including the lipoxin, resolvin, protectin and maresin families could be new therapies[7, 8]. Lipoxins are endogenously generated from arachidonic acid, which are formed principally by transcellular metabolism initiated by sequential oxygenation of arachidonic acid by either 15- and 5-lipoxygenases or 5- and 12-lipoxygenases and exhibit anti-inflammatory proresolution properties. Lipoxin A4 (LXA4) and its positional isomer lipoxin B4 (LXB4) are the principal species formed in mammals. In addition to the classic lipoxin-generating pathways, another recognized pathway of lipoxins biosynthesis is called aspirin-triggered lipid-generating pathway. It is initiated when COX-2 is up-regulated and irreversibly acetylated by aspirin, producing 15-epi-LXA4 and 15-epi-LXB4. LXA4 and 15-epi-LXA4 elicit the multicellular responses via a specific G protein-coupled receptor (GPCR), termed ALXR that is identified in human, mouse and rat tissues. LXB4 does not bind ALXR, and the LXB4 receptor remains to be identified[6, 9]. Therefore, most studies of lipoxins were focused on LXA4.

Current evidence has revealed that LXA4 is involved in the protective mechanism from MetS by attenuating adipose inflammation, and improving insulin sensitivity in animal models. Su. et al reported that plasma LXA4 level was decreased by 120% in rats with MetS which might be responsible for the exaggerated and persistent postoperative cognitive decline[10]. Some researchers reported that specialized proresolving lipid mediators such as LXA4 could resolve inflammation and improve insulin sensitivity[11, 12].

However, to our knowledge, there are currently no available clinical and epidemiological studies that associate serum LXA4 level with the development of MetS. Here, we clarified the relationship between serum LXA4 level and the development of MetS.

## Materials and Methods

### 1. Study subjects

#### 1.1 Cross-sectional study

Beginning in March 2010, 624 eligible Han Chinese adults aged 40–65 years old were enrolled in the Caihe communities of Hangzhou, Zhejiang province, China. The Medical Ethics Committee of Sir Run Run Shaw Hospital affiliated to School of Medicine, Zhejiang University approved this study. All patients gave written informed consent. The following subjects were excluded according to: 1) a previous diagnosis of diabetes, 2) moderate to severe hypertension (BP>160/100 mmHg at rest), 3) impaired liver or renal function, 4) malignant tumors, 5) cardiovascular or peripheral vascular disease, 6) acute infectious disease or chronic inflammatory disease, 7) treatment with acetylsalicylic acid, or nonsteroidal anti-inflammatory drugs (NSAIDS), 8) treatment with lipid-lowering drugs, 9) treatment with Metformin or thiazolidinediones, 10) treatment with angiotensin-converting enzyme inhibitors (ACEI) or angiotensin receptor blocker (ARB), 11) pregnancy.

#### 1.2 Prospective study

All 624 participants who were enrolled in the study in 2010 were contacted for the 2.5-year follow-up study. 74 participants were lost during the follow-up, 126 participants (including 4 participants with MetS at baseline) dropped out the study, 5 participants were dead, 2 participants withdrew from the study for incomplete laboratory data. Total 417 participants were eligible for the 2.5-year follow-up. 333 participates who didn’t meet a diagnosis of Mets at baseline were analyzed in the 2.5-year follow-up study.

#### 1.3 Definition

MetS was defined according to standards generated by the Joint Committee for Developing Chinese Guidelines on Prevention and Treatment of Dyslipidemia in Adults (JCDCG)[13]. Individuals who met three or more of the following criteria were considered as having MetS: central obesity (WC >90 cm for men and >85 cm for women); hypertriglyceridemia (≥1.70 mmol/L); low HDL-c (<1.04 mmol/L); elevated BP (≥130/85 mmHg or current treatment for hypertension); and hyperglycemia [fasting plasma glucose (FPG)≥6.1 mmol/L or 2h postprandial glucose (2h PG)≥7.8 mmol/L].

Current smokers were defined as individuals who smoked at least one cigarette per day for over 6 months[14]. Alcohol drinkers were defined as individuals who consumed alcohol more than 3 days a week[15].

### 2. Measurements

Baseline and follow-up anthropometric and metabolic measures were conducted using standardized methods in the same local community health care center. All participants were interviewed face-to-face by trained medical staff, completing a standardized questionnaire regarding demographic data, life style, present and past illness, medical therapy and other health-related information.

All participants submitted to the local community health care center between 7 and 8 am following an overnight fast. Anthropometric data included measurements of height, weight, waist circumference (WC), hip circumference, heart rate (HR) and blood pressure (BP). Body mass index (BMI) was calculated as weight in kilograms divided by the square of height in meters (kg/m^2^). WC was measured at the midpoint between the lowest rib and the iliac crest and hip circumference was measured at the widest point of the hips in the standing position. Waist–to-hip ratio (WHR) was calculated by dividing WC by hip circumference. Systolic blood pressure (SBP) and diastolic blood pressure (DBP) were calculated as the average of three measurements, using a mercury sphygmomanometer at 3-min intervals. The percent body fat was assessed using a Tanita Body Composition Analyzer TBF-300 (Tanita Corporation, Tokyo, Japan). All subjects underwent abdominal MRI using a whole-body imaging system (SMT-100, Shimadzu Co, Kyoto, Japan) with TR-500 and TE-200 of SE. MRI scans were performed at the level of umbilicus between L4 and L5 with the subject in the supine position. Abdominal visceral fat area (VFA) and abdominal subcutaneous fat area (SFA) were calculated using SliceOmatic software (version 4.2).

All the participants conducted a 75-g oral glucose tolerance test. FPG and 2hPG, triglyceride (TG), total cholesterol (TC), low density lipoprotein-cholesterol (LDL-c), high density lipoprotein-cholesterol (HDL-c) and C-reactive protein (CRP) were measured with an auto-analyzer (Aeroset, Chicago, IL, USA). Serum insulin levels were measured by radioimmunoassay using an insulin detection kit (Beijing North Institute Biological Technology, China). Insulin sensitivity was assessed by homeostasis model assessment for insulin resistance (HOMA-IR) based on fasting glucose and insulin measurements as follows: [insulin (μIU/ml)×fasting blood glucose (mg/dl)/18]/22.5[16]. Glycosylated hemoglobin A _1_c (HbA _1_c) values were tested using ion-exchange high-performance liquid chromatography (Hemglobin Testing System; Bio-Rad, Hercules, CA, USA).

LXA4 was determined via quantitative sandwich ELISA (Cusabio Biotech, Wuhan, China) with intra- and inter-assay coefficients of variation of < 8 and < 10%.

### 3. Statistical analysis

All the continuous variables were tested for normal distribution and normally distributed variables were expressed as mean ± standard deviation (SD). Variables with a skewed distribution were presented as median (interquartile range, 25–75%), which were logarithmically transformed to achieve a normal distribution before analysis. Categorical variables were presented as frequency and percentage. Differences of baseline characteristics of between participants with and without Mets were analyzed by t-test for continuous variables and chi-square test for categorical variables. Correlations between serum LXA4 levels and the metabolic parameters before and after adjustment of age, gender, smoking status, and drinking status were obtained by Pearson correlation analysis in the cross-sectional study. To determine the difference of metabolic parameters at 2.5-year follow-up among three subgroups according to baseline serum LXA4 concentrations, one-way analysis of variances (ANOVAs) was used for continuous variables. The chi-square test was used to compare the prevalence data. The incidence of the MetS at different levels of serum LXA4/WC or LXA4/VFA was analyzed by Chi-squared analysis. Logistic regression analysis was used in the prospective study to test the influences of variables on the risk of MetS. SPSS 20.0 (IBM, Armonk, NY, USA) was used for statistical analysis and the 2-sided P <0.05 was considered statistically significant.

## Results

### Baseline participant characteristics

The anthropometric and metabolic characteristics of the study population at baseline were presented in Table 1. The average age of the participants was 56.27 (±6.85) years, and 35.9% of the participants were male. There was no gender difference in serum LXA4 levels. At baseline, 88/624 (14.1%) of individuals met criteria for MetS.

**Table 1:** Baseline Characteristics of Participants According to the Presence or Absence of Mets.

	Total	Mets absent	Mets	P for trend
**N (%)**	**624 (100)**	**536 (85.9)**	**88 (14.1)**	
**Age (years)**	**56.27±6.85**	**56.15±6.82**	**57.02±7.02**	**0.269**
**Male, n(%)**	**224(35.9)**	**163(30.4)**	**61(69.3)**	**<0.001**
**Current smoker, n(%)**	**140(22.4)**	**91(17.0)**	**49(55.7)**	**<0.001**
**Alcohol drinker, n(%)**	**124(19.9)**	**83(15.5)**	**41(46.6)**	**<0.001**
**BMI(kg/m** ^**2**^ **)**	**23.38±2.97**	**22.94±2.76**	**26.04±2.83**	**<0.001**
**WC (cm)**	**77.90±9.12**	**76.10±8.09**	**88.81±7.18**	**<0.001**
**WHR**	**0.87±0.07**	**0.85±0.07**	**0.95±0.06**	**<0.001**
**Fat% (%)**	**29.39±6.97**	**28.99±6.95**	**31.79±6.64**	**<0.001**
**SBP (mm Hg)**	**121.32±15.45**	**119.32±14.70**	**133.47±14.37**	**<0.001**
**DBP (mm Hg)**	**80.63±12.05**	**78.11±13.04**	**84.79±8.74**	**<0.001**
**FPG (mmol/L)**	**5.02±1.17**	**4.91±0.96**	**5.71±1.89**	**<0.001**
**2h PG (mmol/L)**	**6.15±3.19**	**5.78±2.82**	**8.45±4.21**	**<0.001**
**FINS (μU/ml)**	**10.70(8.18–13.87)**	**10.19(7.97–13.07)**	**14.22(11.18–18.51)**	**<0.001**
**2h INS (μU/ml)**	**57.79(37.32–87.80)**	**54.79(36.39–83.57)**	**81.50(47.75–155.18)**	**<0.001**
**HOMA-IR**	**2.31(1.73–3.13)**	**2.16(1.68–2.87)**	**3.48(2.55–5.26)**	**<0.001**
**HbA1**_**C**_ **(%)**	**5.67±0.68**	**5.61±0.63**	**6.05±0.87**	**<0.001**
**TC (mmol/L)**	**5.62±1.01**	**5.58±1.01**	**5.85±1.02**	**0.018**
**LDL-c (mmol/L)**	**2.42±0.57**	**2.42±0.57**	**2.45±0.58**	**0.607**
**HDL-c (mmol/L)**	**1.47±0.36**	**1.53±0.35**	**1.12±0.25**	**<0.001**
**TG (mmol/L)**	**1.28(0.93–1.78)**	**1.20(0.87–1.55)**	**2.31(1.81–3.26)**	**<0.001**
**CRP (mg/L)**	**0.65(0.29–1.65)**	**0.62(0.28–1.53)**	**1.80(0.98–3.86)**	**0.002**
**SFA (cm** ^**2**^ **)**	**154.60(118.83–202.70)**	**153.95(114.60–201.85)**	**168.55(128.33–206.70)**	**0.008**
**VFA (cm** ^**2**^ **)**	**68.14(44.18–106.78)**	**62.49(39.92–90.59)**	**127.35(102.03–161.65)**	**<0.001**
**LXA4 (pg/ml)**	**79.75(58.83–112.03)**	**81.36(59.62–113.59)**	**72.88(52.03–89.28)**	**0.003**

Values are presented as mean ± SD for normally distributed continuous variables, median (interquartile range) for skewed variables, and number (%) for categorical variables. P for trend indicates the significance in the difference between participants with and without metabolic syndrome. MetS: metabolic syndrome; LXA4: lipoxin A4; BMI: body mass index; WC: waist circumference; WHR: waist–hip ratio; Fat%: body fat percentage; SBP: systolic blood pressure; DBP: diastolic blood pressure; FPG: fasting plasma glucose; 2h PG: 2-hour postprandial glucose; FINS: fasting insulin; 2h INS: 2-hour insulin; HOMA-IR: homeostatic model assessment of insulin resistance; HbA1c: glycosylated hemoglobin A1c; TC: total cholesterol; LDL-c: low density lipoprotein cholesterol; HDL-c: high density lipoprotein cholesterol; TG: triglyceride; CRP: C-reactive protein; SFA: subcutaneous fat tissue area; VFA: visceral fat tissue area.

As expected, participants with MetS had more cardiovascular risk factors at baseline compared to those without MetS, including higher BMI, WHR, Fat%, fasting insulin levels, HOMA-IR, HbA1c, TC, CRP, SFA, VFA, as well as MetS components (higher WC, blood glucose, TG, BP, and lower HDL-c) (P<0.05 for all parameters). Furthermore, participants with MetS had lower serum LXA4 levels compared with those without MetS [72.88(52.03–89.28) vs 81.36(59.62–113.59) pg/ml, P = 0.003].

### Correlations between serum LXA4 and metabolic parameters at baseline

Correlations between serum LXA4 levels and metabolic parameters were conducted (Table 2). Serum LXA4 levels were negatively correlated with BMI, WC, WHR, FPG, HbA1c, LDL-c, SFA, and most significantly with VFA (r = -0.162, P<0.001)(all P<0.05). After controlling for the confounding factors of age, gender, smoking and drinking status, the inverse relationships between serum LXA4 levels and BMI, WC, WHR, SFA and VFA remained, with the strongest correlation found between baseline LXA4 and VFA (r = -0.143, P = 0.001).

**Table 2:** Correlations Between Serum LXA4 and Metabolic Parameters at Baseline (n = 624).

	Unadjusted		Age, sex, smoking, and drinking adjusted	
	r	P value	r	P value
**BMI(kg/m** ^**2**^ **)**	**-0.098**	**0.015**	**-0.079**	**0.050**
**WC (cm)**	**-0.134**	**0.001**	**-0.102**	**0.012**
**WHR**	**-0.114**	**0.004**	**-0.082**	**0.042**
**Fat% (%)**	**-0.038**	**0.346**	**-0.050**	**0.219**
**SBP (mm Hg)**	**-0.054**	**0.178**	**-0.010**	**0.810**
**DBP (mm Hg)**	**-0.013**	**0.740**	**0.026**	**0.520**
**FPG (mmol/L)**	**-0.055**	**0.033**	**-0.066**	**0.103**
**2h PG (mmol/L)**	**-0.076**	**0.059**	**-0.054**	**0.184**
**FINS (μU/ml)**	**0.030**	**0.496**	**0.037**	**0.396**
**2h INS (μU/ml)**	**-0.022**	**0.618**	**-0.020**	**0.642**
**HOMA-IR**	**-0.013**	**0.748**	**0.001**	**0.989**
**HbA1**_**C**_ **(%)**	**-0.089**	**0.026**	**-0.063**	**0.119**
**TC (mmol/L)**	**-0.052**	**0.232**	**-0.046**	**0.257**
**LDL-c (mmol/L)**	**-0.079**	**0.050**	**-0.067**	**0.095**
**HDL-c (mmol/L)**	**0.011**	**0.789**	**0.008**	**0.842**
**TG (mmol/L)**	**-0.070**	**0.079**	**-0.027**	**0.535**
**CRP (mg/dL)**	**-0.011**	**0.859**	**0.009**	**0.881**
**SFA (cm** ^**2**^ **)**	**-0.086**	**0.048**	**-0.085**	**0.050**
**VFA (cm** ^**2**^ **)**	**-0.162**	**<0.001**	**-0.143**	**0.001**

Abbreviations as in Table 1.

### Difference of metabolic parameters at 2.5-year follow-up among subgroups according to baseline serum LXA4 concentrations

In the prospective study, 333 participants of 417 eligible participants (84 participants who met MetS criteria at baseline were excluded) were included for analysis. Among them, 32(9.6%) developed MetS over the 2.5-year follow-up. All 333 participants were categorized into 3 groups based on tertile of serum LXA4 levels and characteristics of participants were shown in table 3. Serum LXA4 levels in different tertiles were 50.64(40.21–62.42), 87.07(76.39–95.44), and 130.08(117.50–161.31) pg/ml. A decrease in serum LXA4 levels at baseline was accompanied by an increase in new incidence of MetS (P = 0.072). A higher incidence of new Mets was found in the participants of the lowest tertile as compared with that in participants of the highest tertile(P = 0.025).

**Table 3:** Association of Baseline LXA4 Concentrations with Metabolic Parameters at 2.5-year Follow-up.

Variables	T1	T2	T3	P for trend
**n**	**111**	**111**	**111**	
**LXA4 (pg/ml)**	**50.64(40.21–62.42)**	**87.07(76.39–95.44)**	**130.08(117.50–161.31)**	**<0.001**
**new Mets cases(%)**	**16(14.4)**	**10(9.0)**	**6(5.4)**	**0.072**
**Age (years)**	**56.38±6.85**	**54.87±6.72**	**56.03±6.10**	**0.201**
**Male, n(%)**	**34(30.6)**	**30(27.0)**	**33(29.7)**	**0.828**
**Current smoker, n(%)**	**20(18.0)**	**20(18.0)**	**11(9.9)**	**0.153**
**Alcohol drinker, n(%)**	**29(26.1)**	**16(14.4)**	**10(9.0)**	**0.002**
**BMI(kg/m** ^**2**^ **)**	**24.05±2.54**	**23.51±2.93**	**23.34±2.33**	**0.108**
**WC (cm)**	**81.08±8.80**	**78.45±9.09**	**78.17±7.23**	**0.019**
**WHR**	**0.88±0.07**	**0.86±0.08**	**0.86±0.07**	**0.032**
**Fat% (%)**	**30.52±6.77**	**30.36±8.17**	**28.78±6.84**	**0.148**
**SBP (mm Hg)**	**122.52±13.56**	**123.27±14.42**	**121.26±13.27**	**0.548**
**DBP (mm Hg)**	**74.41±9.50**	**74.58±8.82**	**72.79±8.61**	**0.262**
**FPG (mmol/L)**	**5.30±0.68**	**5.18±0.68**	**5.20±0.70**	**0.425**
**2h PG (mmol/L)**	**6.87±2.85**	**5.67±2.62**	**6.04±2.90**	**0.005**
**FINS (μU/ml)**	**7.11(5.07–9.95)**	**7.12(5.22–9.89)**	**7.11(4.98–8.75)**	**0.172**
**2h INS (μU/ml)**	**35.30(21.07–59.72)**	**28.27(16.91–51.16)**	**31.74(21.83–47.69)**	**0.094**
**HOMA-IR**	**1.62(1.16–2.42)**	**1.63(1.20–2.28)**	**1.65(1.08–2.07)**	**0.159**
**HbA1**_**C**_ **(%)**	**5.53±0.75**	**5.51±0.59**	**5.45±0.73**	**0.684**
**TC (mmol/L)**	**5.14±1.01**	**5.06±0.91**	**4.93±1.12**	**0.281**
**LDL-c (mmol/L)**	**3.22±0.89**	**3.17±0.76**	**3.03±0.96**	**0.227**
**HDL-c (mmol/L)**	**1.38±0.31**	**1.32±0.28**	**1.34±0.30**	**0.399**
**TG (mmol/L)**	**1.20(0.90–1.53)**	**1.20(0.90–1.60)**	**1.20(0.90–1.50)**	**0.641**
**SFA (cm** ^**2**^ **)**	**162.20(121.90–205.80)**	**154.40(112.30–201.90)**	**152.20(107.30–202.40)**	**0.377**
**VFA (cm** ^**2**^ **)**	**68.55(48.85–106.80)**	**61.28(36.20–79.89)**	**58.74(35.87–76.41)**	**0.005**

Abbreviations as in Table 1.

### Risk of MetS at 2.5-year follow-up relative to baseline LXA4 concentrations, WC and VFA

Because abdominal obesity plays an important role in the development of MetS and we have found a close relationship between VFA/WC and serum LXA4 levels, next, we further assessed whether a low level of serum LXA4 could also effectively predict the risk of incident MetS. Logistic regression analyses were conducted after participants were categorized by median plasma LXA4 levels, WC based on cutpoints recommended for central obesity by IDF (WC≥90cm for male or WC≥80cm for female) [17] and VFA based on cutpoints using 80cm^2^[18](Table 4). After adjustment by confounding factors of age, gender, smoking and drinking status, there was a higher risk of incident MetS in participants with low LXA4 levels compared with those with high LXA4 levels (HR 2.607 [95% CI 1.151–5.909], P = 0.022), as well as in those with high VFA versus with those with low VFA (HR 2.571 [95% CI 1.176–5.620], P = 0.018). However, the risk of incident MetS in participants with high WC was not statistically different from those with low WC (HR 2.106 [95% CI 0.905–4.900], P = 0.084).

**Table 4:** Risk of MetS at 2.5-year Follow-up Relative to Baseline LXA4 Concentrations, WC and VFA.

Variables		new Mets Cases/number of participants(%)	ExpB (95% CI)	P value
**LXA4**	**LXA4<87.07 pg/ml**	**23/167 (14%)**	**2.607(1.151–5.909)**	**0.022**
	**LXA4≥87.07 pg/ml**	**9/166 (5%)**	**1(reference)**	
**WC**	**WC≥90cm for male or WC≥80cm for female**	**11/71 (15%)**	**2.106 (0.905–4.900)**	**0.084**
	**WC<90cm for male or WC<80cm for female**	**21/262 (8%)**	**1(reference)**	
**VFA**	**VFA≥80cm** ^**2**^	**16/93(17%)**	**2.571(1.176–5.620)**	**0.018**
	**VFA<80cm** ^**2**^	**16/240(7%)**	**1(reference)**	

Adjusted for age, sex, current smoking, alcohol drinking.

Abbreviations as in Table 1.

### Risk of MetS at 2.5-year follow-up relative to the combination of baseline LXA4 concentrations and WC/VFA

To determine whether a combination of serum LXA4 levels and WC can better predict the risk of MetS, participants were classified into four groups based on their levels of LXA4 and WC: 1) low LXA4/high WC, 2) low LXA4/low WC, 3) high LXA4/high WC, 4) high LXA4/low WC. The incidence of MetS in participants with a low LXA4 and high WC was highest among the four groups at 2.5-year follow-up, with 4.897 times higher risk of incident MetS (HR 4.897 [95% CI 1.485–16.147], P = 0.009) versus those with high LXA4/low WC after adjustment for age, gender, smoking and drinking status(Table 5).

**Table 5:** Risk of MetS at 2.5-year Follow-up Relative to the Combination of Baseline LXA4 Concentrations and WC.

LXA4	WC	new Mets Cases	ExpB (95% CI)	P value
**Low**	**High**	**8/42(19%)**	**4.897 (1.485–16.147)**	**0.009**
**Low**	**Low**	**15/126(12%)**	**1.784 (0.423–7.522)**	**0.431**
**High**	**High**	**3/29(10%)**	**1.747 (0.646–4.723)**	**0.272**
**High**	**Low**	**6/136(4%)**	**1**	

Adjusted for age, sex, current smoking, alcohol drinking.

Definition of high or low LXA4/WC as in table 4.

Abbreviations as in Table 1.

When participants were classified into four groups according to the combination of serum LXA4 levels and VFA (table 6), risk of MetS among the four groups showed the similar trend to the results described above: the incidence of MetS in participants with a low LXA4 and high VFA was highest among the four groups at 2.5-year follow-up, with 4.967 times higher risk of MetS (HR 4.967 [95% CI 1.705–14.475], P = 0.003) versus those with high LXA4/low VFA after adjustment for age, gender, smoking and drinking status.

**Table 6:** Risk of MetS at 2.5-year Follow-up Relative to the Combination of Baseline LXA4 Concentrations and VFA.

LXA4	VFA	new Mets Cases	ExpB (95% CI)	P value
**Low**	**High**	**13/63(21%)**	**4.967 (1.705–14.475)**	**0.003**
**Low**	**Low**	**10/105(10%)**	**2.336 (0.592–9.222)**	**0.226**
**High**	**High**	**3/30(10%)**	**2.291 (0.895–5.865)**	**0.084**
**High**	**Low**	**6/135(4%)**	**1**	

Adjusted for age, sex, current smoking, alcohol drinking.

Definition of high or low LXA4/VFA as in table 4.

Abbreviations as in Table 1.

## Discussion

In recent years, lipoxins (LXs) and resolvins (Rvs) have attracted intense interest because of increasing evidence of their involvements, particularly in chronic disorders where unresolved inflammation is a central factor. Some previous studies indicated that LXA4 may have favorable effects in patients with MetS or T2DM[11, 19]. In our cross-sectional study we demonstrated an inverse association between serum LXA4 levels and prevalence of MetS as well as the following indexes related to obesity including BMI, WC, WHR, SFA and VFA in Chinese middle-aged population. Among 333 participants who had not been diagnosed as MetS at baseline, 32(9.6%) developed MetS at 2.5-year follow-up. The risk of development to MetS was 2.607 times higher for participants with lower serum LXA4 levels than those with higher serum LXA4 levels. When we classified the participants according to the combination of serum LXA4 level and WC/VFA, we found that the risk of development to MetS was 4.897/4.967 times higher for participants in the low LXA4 and high WC/VFA subgroup than those in the high LXA4 and low WC/VFA subgroup.

Although multiple molecular mechanisms likely underlie obesity and its complications, low-graded, chronic inflammation in fat tissue is known to be critically involved in the pathophysiology of insulin resistance which is the key step to develop MetS and T2DM[20, 21]. Obesity causes a phenotypic switch from the M2 macrophages to M1 phenotype, correlating with insulin resistance both in mice and humans[22]. Direct and paracrine signals issued from M1 macrophages can impair insulin signaling and adipogenesis in adipocytes whereas M2 macrophages seem to protect against obesity-induced insulin resistance. LXA4 is able to enhance M2 macrophage polarization[23]. Thus, a deficiency in LXA4 may keep the chronic inflammatory state in adipose tissue and induce insulin resistance.

Accumulating evidence suggested a number of proinflammatory factors, such as chemerin, TNF-α, IL-1β, and IL-6, were involved in the development of obesity related disorders[24–27]. There is evidence to indicate that TNF-αand IL-6 may interfere with the metabolism of essential fat acids (EFAs)[28, 29] and thus, induce a deficiency of the formation of precursors of lipoxins, resolvins, and protectins. On the other hand, these lipoxins, resolvins, and protectins have the ability to suppress the production of IL-6 and TNF-α[30]. When this balance is impaired, enhanced production of IL-6 and TNF-α will lead to IR.

Recently, Demetz et al reported that LXA4, 15-epi-LXA4, and LXB4 increased hepatic Abcb11 protein expression in mice, which could enhance reverse cholesterol transport (RCT), a process in which transfer of cholesterol to high-density lipoprotein (HDL) enables the return of excess cholesterol to the liver for excretion in the form of bile acids[31]. In our study, we found serum LXA4 levels were negatively correlated with LDL-c(p = 0.05), but the relation was not significant after adjustment(P = 0.095). It maybe needs a larger population to confirm this view.

Visceral adipose tissue is considered to have higher inflammatory responses than subcutaneous tissue[32–34], and accumulation of visceral adipose has been defined as a more valuable risk factor than other obesity related indices for development of MetS, diabetes and CVD[35–38]. Thus a combination of indices of both obesity and inflammation could have more powerful predictive value. We hypothesized that a combination of LXA4 and WC/VFA would be more powerful to predict the risk of MetS than LXA4/WC/VFA alone. As expected, the risk of development to MetS was 4.897/4.967 times higher for participants in the low LXA4 and high WC/VFA subgroup than those in the high LXA4 and low WC/VFA subgroup. The predictive power was apparently enhanced compared with using LXA4 (2.607 times), WC(2.106 times) or VFA(2.571 times) alone. A combination of LXA4 and WC presented a similar powerful predictive value for the development of MetS to the combination of LXA4 and VFA. Furthermore, assessment of LXA4 and WC is more convenient and more economical than measuring VFA through MRI or CT.

Until now the knowledge on clinical and epidemiological studies on the role of LXA4 in obesity and metabolic disorders is scarce. Pickens et al showed the concentration of LXA4 increased in the obese subjects[39], but we observed the opposite association. A number of factors can possibly explain the discrepancies. First, the participants were recruited from different racial background. Second, their results were expressed as fold changes between concentrations of LXA4 in the obese to lean plasma pools, which is different from our methods. Third, they investigated only 10 participants from the obese and lean group respectively to assess the correlation between serum LXA4 levels and obesity. However, Kaviarasan et al recently reported a progressive decrease of LXA4 levels with 34% fall in T2DM[40], consistent with our report. Nonetheless, further studies are warranted to clarify these findings.

The present study has limitations may affect the interpretation of our results. For instance, the absolute amount of dietary AA may influence serum LXA4 levels, and we did not collect correlative dietary information in this study. However, given that Han Chinese in the same district usually have a similar dietary habit, we consider dietary effect is likely very limited. Because of the relatively short follow-up time of 2.5 years and loss of subjects during follow-up, the number of participants who developed MetS was relatively small. Since we focused on a mid-aged Chinese population, our findings should be applied to other demographic groups with caution. Finally, the exact protective mechanisms in which LXA4 is involved need to be further studied. Despite these limitations, this is the first epidemiological study that associates LXA4 to MetS. It has recently been shown that supplemented with n-3 fat acids(FAs) could prevent LXA4 levels from decreasing in the culture medium of peripheral blood mononuclear cells from Alzheimer's disease patients[41], indicating LXA4 can potentially be used as a therapeutic target.

## Conclusions

In conclusion, our findings suggest LXA4 could be a protective factor and its decrease in serum might be a predictive biomarker of obesity-associated inflammation and early MetS. Thus, LXA4 might serve as a new therapeutic target for regulation of inflammation in adipose tissue and for protection of obesity related diseases. Finally, we show that a combination of LXA4/WC or LXA4/VFA is able to powerfully predict the development of MetS.

## Supporting Information

S1 Table Data of Participants at Baseline. (XLS)

S2 Table Data of Participants at 2.5-year Follow-up. (XLS)

S3 Table Logistic analysis showing variables independently associated with prevalence of MetS. Variables included are as follows: gender, age, smoking status, drinking status, LXA4, VFA, BMI, WC, WHR, FPG, HbA1c, LDL-c, SFA.
(DOC)
