# Supplementary material for: Inverse Relationship between Serum Lipoxin A4 Level and the Risk of Metabolic Syndrome in a Middle-Aged Chinese Population
Source: PLoS One. 2015 Nov 13;10(11):e0142848. doi: 10.1371/journal.pone.0142848 (PMC4643896; doi:10.1371/journal.pone.0142848)
Supplement: S3 Table — Variables included are as follows: gender, age, smoking status, drinking status, LXA4, VFA, BMI, WC, WHR, FPG, HbA1c, LDL-c, SFA. (DOC) [file pone.0142848.s003.doc]

**S3 Table.** **Logistic analysis showing variables independently associated with** **prevalence of MetS.**

| **Variables** | **EXP(B) (95% CI)** | **P** |
| --- | --- | --- |
| **LXA4** | **0.15(0.03-0.83)** | **0.029** |
| **VFA** | **18.20(2.60-127.52)** | **0.003** |
| **FPG** | **1.35(1.03-1.78)** | **0.032** |
| **HDL-c** | **0.19(0.05-0.78)** | **0.021** |

**Variables included are as follows: gender, age, smoking status, drinking status, LXA4, VFA, SFA , BMI, WC, WHR, FPG, HbA1c, LDL-c, HDL-c.**

**MetS: metabolic syndrome; LXA4: lipoxin A4; VFA: visceral fat tissue area; SFA: subcutaneous fat tissue area; BMI: body mass index; WC: waist circumference; WHR: waist–hip ratio; FPG: fasting plasma glucose; HbA1c: glycosylated hemoglobin A1c; LDL-c: low density lipoprotein cholesterol; HDL-c:** **high density lipoprotein cholesterol.**
